# Preliminary Findings on the Unified Protocol for Transdiagnostic Treatment: A Pilot Trial on Its Effectiveness on Anxiety and Depressive Symptoms for Turkish Adults

**DOI:** 10.1002/jclp.70126

**Published:** 2026-03-12

**Authors:** Özge Erarslan‐İngeç, Orçun Yorulmaz, Todd J. Farchione, Madison Boschulte

**Affiliations:** ^1^ Department of Psychology Dokuz Eylul University Izmir Turkey; ^2^ The Center for Anxiety and Related Disorders Boston University Boston Massachusetts USA

**Keywords:** anxiety disorders, depression, transdiagnostic approach, Unified Protocol for Transdiagnostic Treatment of Emotional Disorders

## Abstract

The present study investigates the feasibility and initial efficacy of the Unified Protocol (UP) as a pilot trial for anxiety and depressive symptoms in Turkish university students with a pretest‐posttest control group research design. Thirty‐four undergraduate students with high levels of depressive and/or anxiety symptoms were randomly assigned to one of two study conditions: an intervention group based on the UP (*n* = 17) or a waitlist control group (*n* = 17). To determine the effectiveness of treatment, a 2 (intervention‐control group) x 2 (pre‐post test) mixed measures variance analysis was applied for the primary outcome measures: degree of anxiety and depressive symptoms as well as positive and negative affect and psychological well‐being. The analyses revealed a significant difference between pre‐ and post‐treatment scores on all outcome measures in the intervention group; these improvements were also significantly greater than those in the control group. These outcomes included participants receiving the Unified Protocol reported lower levels of anxiety and depressive symptoms, decreased negative affect, higher levels of positive affect, and improved psychological well‐being compared to the control condition. The findings of this study provide preliminary support for the efficacy of the Unified Protocol in a Turkish sample. The study findings are evaluated in light of the relevant literature and within the scope of the study's limitations, and suggestions for academic and clinical applications are presented.

Anxiety and mood disorders are the most common mental health problems, with lifetime prevalence rates estimated at 29% and 21% of the United States population, respectively (Kessler et al. 2005). In addition, both have a high rate of medical and psychiatric comorbidity (Kessler et al. 2005). Comorbidity between anxiety and mood disorders is further associated with more severe problems, decreased functioning, more frequent relapse, higher risk of suicide, and greater utilization of health services (Lamers et al. 2011).

Cognitive Behavioral Therapy (CBT) is an effective treatment for anxiety and mood disorders (Hofmann et al. 2012). Although effective, disorder specific CBT protocols require clinicians to master multiple manuals, which increases training demands and potentially complicates treatment planning for clients with comorbid conditions (Castro‐Camacho et al. 2023). Recent findings suggest that transdiagnostic treatments may be an effective alternative to diagnostic‐specific manuals with the advantage of simultaneously treating comorbidity by targeting shared underlying psychopathologic features rather than surface‐level DSM symptoms (Bullis et al. 2019). The Unified Protocol for Transdiagnostic Treatment of Emotional Disorders (UP; Barlow et al. 2017) is one such treatment that has garnered considerable evidence for its use. The UP was explicitly designed to address dysfunction in the interpretation and regulation of emotion associated with neuroticism and is believed to contribute to the onset and maintenance of what Barlow and colleagues have described as “emotional disorders” (Bullis et al. 2019). The foundation of the UP is that neuroticism and related emotion dysregulation are core features underlying emotional disorders, including anxiety, obsessive‐compulsive, and depressive disorders. The UP consists of eight treatment modules: motivation enhancement, psychoeducation of emotional experiences, emotion awareness training, cognitive appraisal and reappraisal, emotion avoidance and emotion‐driven behaviors, awareness and tolerance training for physical sensations, emotion exposures, and relapse prevention. The modules comprise emotion‐focused cognitive behavioral strategies for adaptive emotion regulation, including mindful awareness of emotions, cognitive flexibility, changing maladaptive behaviors, understanding and confronting physical sensations, and emotion exposures (Farchione et al. 2012).

Since its development, the UP's efficacy has been rigorously tested in heterogeneous sample groups seeking treatment for anxiety, depression, and related disorders. Initially, the UP was tested in a pilot trial of individuals with anxiety disorders at the Center for Anxiety and Related Disorders (CARD) at Boston University (Ellard et al. 2010). Farchione et al. (2012) then conducted an initial randomized controlled trial with 37 participants diagnosed with heterogeneous anxiety disorders. Results indicated that participants in the treatment group had significant reductions in anxiety symptoms compared to those in the waitlist control group. Barlow et al. (2017) followed up on these promising findings by conducting a large (*N* = 223) randomized, non‐inferiority, controlled trial comparing the UP to well‐established CBT protocols focusing on a single disorder (SDP). In that study, treatment‐seeking adults diagnosed with generalized anxiety disorder, panic disorder, social anxiety disorder or obsessive‐compulsive disorder were randomly assigned to three conditions: UP treatment (*n* = 88), diagnosis‐specific treatment (*n* = 91), and waitlist control (*n* = 44). The primary outcome measure for the study was ADIS (Anxiety Disorders Interview Schedule) CSR (clinical severity rating) ratings. The equivalence margin was determined a difference of 0.75 CSR units based on previous CBT outcome studies (i.e. Hoffman and Smits 2008). The UP was found to be equally effective as the single‐disorder treatment protocols, and participants in both treatment groups showed a significant improvement in their principal diagnosis compared to those in the waitlist condition. It was also determined that a greater proportion of participants in the UP condition completed the full treatment protocol (87.5%) compared to those assigned to the single disorder protocols (69.2%).

A recent systematic review and meta‐analysis involving 15 studies (*n* = 1244) based on both individual and group formats showed the effectiveness of the UP on anxiety disorders and depression (Sakiris and Berle 2019). The UP has been adapted (Ametaj et al. 2018) and shown to be effective across different cultures such as Japan (*n* = 104, as a randomized controlled trial, Ito et al. 2023), Brazil (*n* = 48, in a group format as a clinical trial, de Ornelas Maia et al. 2015), Spain (*n* = 220, as a randomized and controlled clinical trial using a group format, Osma et al. 2018), Iran (*n* = 24, as a clinical trial, Mohammadpour et al. 2018), Hong Kong (*n* = 31, as a pilot trial, Powell et al. 2021) and Colombia (*n* = 200, as a randomized clinical trial, Castro‐Camacho et al. 2023).

The studies examining the effectiveness of the UP outside of Western culture have yielded promising results for Turkey. It is believed that UP would adress a significant gap in Turkey, as there is no transdiagnostic and comprehensive intervention program in the country so far, and it offers significant clinical advantages such as increasing access to psychological interventions, reducing the training burden of the therapist, and adressing multiple structures simultaneously (Newby et al. 2015).

Given the evidence supporting the UP's effectiveness and ecological validity and considering the absence of a similar transdiagnostic intervention in Turkish culture, we developed a Turkish adaptation of the UP. The present study seeks to evaluate whether this adaptation can improve anxiety and depressive symptoms in Turkish university students based on the comparison of a treatment group and a waitlist control group (WL). Accordingly, it was hypothesized that participants in the immediate UP treatment condition would have lower levels of anxiety and depressive symptoms compared to those in the waitlist control. It was also expected that the improvements in severity and symptoms of anxiety and depression would be maintained at a 3‐month post‐treatment follow‐up.

## 1 Method

### 1.1 Participants

Participants were recruited from Dokuz Eylul University using stratified purposeful sampling via various digital and paper announcements that specifically searched for university students with anxiety symptoms, depressive symptoms, or both. These symptoms were assessed using self‐reported measurement tools and structured diagnostic assessment was not used to facilitate participation in the study. Individuals were eligible to participate if they (1) presented with symptoms of anxiety and depression as indicated by a score of 14 or above on the Beck Depression Inventory (2) were able to regularly attend weekly treatment sessions, and (3) did not plan to change any current psychotropic medication during the study. Study exclusions consisted primarily of conditions that would require immediate intervention, including psychotic or dissociative symptoms, suicide risk, and substance use, as determined by the independent psychiatrists from the study in advance. Individuals were also excluded if they were students in the psychology department, were already enrolled in psychotherapy, had a personality disorder, or were unable to attend weekly treatment sessions and other study procedures. Information regarding these exclusion criteria was sent to the clients via an e‐mail form before the random assignment. The present study sample comprised 34 participants, of whom 17 were women (50%). The mean age in the sample was 22.41 years (SD = 1.54, range = 20–26). Fourteen of 34 participants reported that they had received a psychiatric diagnosis such as an anxiety disorder, depressive disorder, obsessive‐compulsive disorder, or post‐traumatic stress disorder at some point in their lives. Except for one person, all others reported that their diagnosis was ongoing at study enrollment. Seven participants (20.6%) were concurrently receiving psychiatric medication. Participants who used medication continued to use it at the same level until the end of the sessions, and confirmation was obtained by the therapist at each session. The distribution of sociodemographic characteristics in the groups is presented in Table 1.

**Table 1:** The characteristics of the participants (*N* = 34).

	UP		Waitlist		Statistics	
	*n*	M (sd)	*n*	M (sd)	t (df)	*p*
Age	17	22.59 (1.37)	17	22.23 ± 1.71	0.66 (32)	0.51
Gender	*n*	%	*n*	%	X2 (df)	*p*
Woman	9	52.9	8	47.1	12 (1).50	
Man	8	47.1	9	52.9		
	*n*	%	*n*	%	X2 (df)	*p*
Diagnosis	8	47.1	6	35.3	0.49 (1)	0.36
Depression	3	17.6	1	5.9		
Anxiety disorder	2	11.8	1	5.9		
OCD	0	—	1	5.9		
ADHD	1	5.9	1	5.9	6.53(8)	0.59
Social phobia	0	—	1	5.9		
Social phobia & depression	0	—	1	5.9		
Panic disorder	1	5.9	0	—		
PTSD	1	5.9	0	—		
	*n*	%	*N*	%	X2 (df)	*p*
Drug treatment	4	23.5	3	17.6	0.18 (1)	0.50

*Note:* *OCD‐Obsessive Compulsive Disorder; ADHD‐Attention Deficit and Hyperactivite Disorder, PTSD‐Post Traumatic Stress Disorder (These diagnostic status were written based on the participants′ self reports).

### 1.2 Measures

*Beck Depression Inventory (BDI):* This inventory—developed by Beck evaluates the somatic, emotional, cognitive, and motivational symptoms of depression. Each one of the 21‐item inventory is scored between 0 and 3. The lowest score that can be obtained from the inventory is 0, and the highest score is 63. The Turkish adaptation study was carried out by Hisli (1988; 1989) based on BDI‐I, and the inventory's Cronbach Alpha internal consistency coefficient was found to be 0.86.

*Beck Anxiety Inventory (BAI):* The BAI was developed by Beck et al. (1988) to evaluate the frequency of anxiety symptoms experienced by individuals. Each item of the 21‐item inventory is scored between 0 and 3, with the highest score being 63. High scores indicate high anxiety symptoms. The Turkish adaptation of the inventory was conducted by Ulusoy et al. (1998) with a clinical sample and the inventory's internal consistency coefficient was reported as 0.93.

*The Overall Anxiety Severity and Impairment Scale (OASIS):* The OASIS is a 5‐item measurement tool developed by Norman et al. (2006) to evaluate the severity and frequency of anxiety symptoms, avoidance behaviors, and functional impairment. The scores that can be obtained from the scale range from 0 to 20. High scores indicate greater severity of symptoms and further deterioration in functioning. In the study in which the scale was developed and its psychometric properties were examined with university students (Norman et al. 2006), the Cronbach Alpha internal consistency coefficient of the scale was 0.80; test‐retest reliability was reported to be 0.82 (1 month time period). For this study, the Turkish translation of the original was used to evaluate anxiety‐related severity and functional impairment in the intervention group. The Cronbach Alpha coefficient of the Turkish version of OASIS was calculated as 0.89 (ERARSLAN İNGEÇ and Yorulmaz 2024).

*The Overall Depression Severity and Impairment Scale (ODSIS):*The ODSIS was developed by adapting the OASIS items to evaluate the severity of depression and its effect on functionality (Bentley et al. 2014). Higher scores indicate more severe depressive symptoms and worsening functioning. The Cronbach's Alpha coefficient of the original scale was found to be 0.94 in the clinical sample, 0.91 in the university students, and 0.92 in the general sample (Bentley et al. 2014). The ODSIS also demonstrated good convergent and discriminant validity. For this study, the Turkish translation of the original was used to evaluate depression‐related severity and functional impairment in the intervention group. The Cronbach Alpha reliability coefficient in a Turkish adaptation study was found to be 0.93 (ERARSLAN İNGEÇ and Yorulmaz 2024).

*The Positive Affect and Negative Affect Scale (PANAS):* The PANAS was developed by Watson et al. (1988) to examine 10 positive and 10 negative emotions. The scores obtained from this scale range from 10 to 50. The internal consistency coefficient of the original scale was found to be 0.88 for positive affect and 0.85 for negative domain. The Turkish adaptation study of the PANAS was carried out by Gençöz (2000). As a result of the factor analysis, it was determined that the items were mainly gathered under positive and negative affect factors as in the original form. The scale's internal consistency was 0.83 for positive affect and 0.86 for negative affect. The test‐retest consistency was found to be 0.40 and 0.54 for positive and negative affect, respectively, consistent with the original study (3 weeks time period). The PANAS was used in the current study to evaluate the effect of the intervention on positive and negative affect.

*Psychological Well‐being (PWB) scale:* The PWB, developed by Diener et al. (2009), consists of eight items related to psychological well‐being, such as positive relationships, a sense of efficacy, and purpose and meaning. High scores obtained from the scale mean that the person has a higher level of psychological well‐being. The internal consistency coefficient of the single‐factor scale was reported as 0.87 in the validity and reliability study conducted with university students. It was reported that the Cronbach Alpha internal consistency coefficient of the Turkish version of the scale was 0.80, and the test‐retest reliability coefficient (2 weeks time period) was 0.86 (Telef 2013).

## 2 Procedure

The Unified Protocol for Transdiagnostic Treatment of Emotional Disorders (UP; Barlow et al. 2011) was adapted into Turkish with permission from the treatment developers from January 2019 to April 2019. After the initial examination by the principal researcher, the second author further evaluated the adaptations of the therapist guide and patient workbook. Individual pilot sessions were conducted with two university students to familiarize the session process and materials to be used, and to finalize the protocol content. During individual sessions, feedback was received from the participants regarding each session and the comprehensibility of the therapy materials. At the end of the process, the final version of the intervention program was created. The Turkish adaptation of UP maintains the eight UP modules; further, it includes much of the original content as well as general treatment format and procedures. Some small revisions were made to facilitate understanding of treatment concepts among Turkish people. For instance, Module 1, which focuses on setting goals and maintaining motivation, was culturally adapted to include barriers to getting help because of the high stigmatization of mental problems in Turkish society. It is known that individuals living in American culture, where the protocol was designed, are exposed to less stigma on mental problems as compared to individuals living in Turkish culture (i.e., Ikizer et al. 2018). In this context, social corcerns about getting therapy were particularly addressed in the first session. Participants who are deeply concerned have also been reminded of confidentiality.

In Module 2 on understanding emotions, the participants' examples were used, and some myths in host culture (e.g., I should feel only positive emotions) were rewritten to develop greater awareness of the adaptive role of emotions. In this module, three‐component model of emotion regulation was discussed with clients' own emotional experiences. The first example of the model that she/he would follow and take notes on throughout the week was made during the session. Among other salient examples of adaptation, there are changes to the music recommended in module 3 to promote mindful emotion awareness. The conscious awareness exercise, which they could use outside the session, was conveyed to the clients via the therapist's voice recording. While the original UP has two core thinking traps_jumping to conclusions and catastrophizing_ in module 4, more thinking traps such as overgeneralization and minimazition/maximitation were included.

The other adaptation was implementation of culturally relevant expressions about the avoidance behaviors in module 5. Especially, oversleeping or skipping exams can be common avoidance issues among university students. These examples were used in the session in detail. In module 6, all participants were informed and exercises were exemplified during the session. However, the homework assignments in this module was skipped if the client was not experiencing severe anxiety. Lastly, some facilitative explanations regarding the emotion exposure hierarchy in module 7 were added, thus helping all participants to create a hierarchy by exemplifying their own experiences that they had mentioned in previous sessions.

After all of the changes had been implemented, the Turkish UP was administered to two participants to check the language and content of the manual. Feedback was solicited to identify potential problems with the intelligibility. Advertising for the study was conducted via social media platforms—including the websites of Dokuz Eylul University's student organizations—and posters were placed in several locations on the university campus. A total of 89 participants expressed interest in the study. Seventeen of them were excluded due to not having significant depressive symptoms—as indicated by a score of 14 or above on the BDI. In addition, 21 participants who did not accept the possibility of being assigned to the waitlist control group or reported barriers to attending weekly sessions were not included in randomization and were referred to the university's medical center. Further, 14 participants reported having active psychiatric conditions that required immediate treatment or could interfere with the study treatment (i.e., three patients with active suicidal thoughts, 1 with psychotic symptoms, 1 diagnosed with borderline personality disorder, 1 diagnosed with dissociative disorder, 5 were engaged in another psychotherapy, and 3 with substance use disorder), so they were also excluded from the study before randomization and directed to the relevant health center. Three students in the psychology department were excluded from the study to prevent possible confounding effects because two researchers work there. They were also informed that they could apply to the medical center at the university if needed.

**Figure 1:**
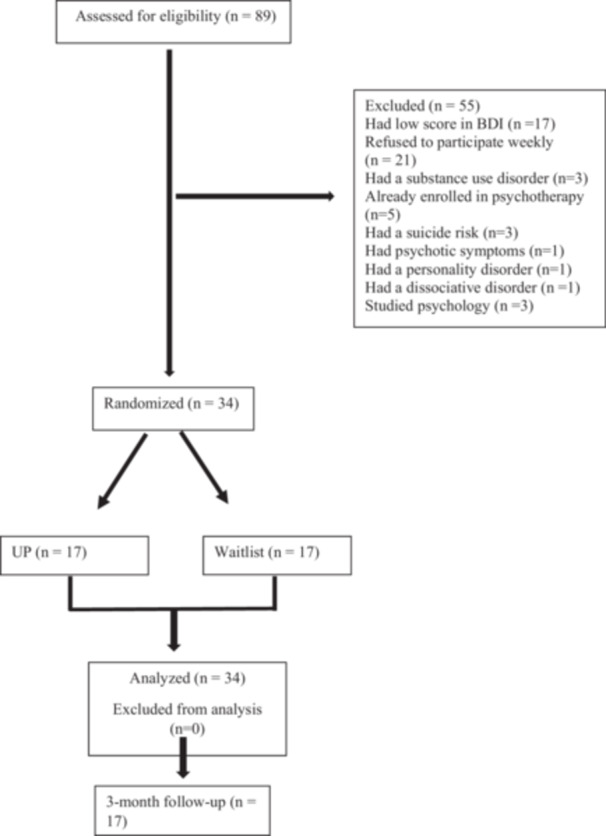
CONSORT diagram illustrating participant flow during the study.

The main study included 34 undergraduate students at Dokuz Eylul University with high levels of anxiety and/or depressive complaints. They were randomly assigned to two conditions: the intervention group (*n* = 17) or a waitlist control group (*n* = 17) using the “simple randomization method”. The assignment process was carried out by an independent researcher using the website https://www.random.org/web and two groups of 17 participants each were created. The decision as to which of these groups would be the experimental group and which would be the control group was made by drawing lots. Figure 1 presents the flow chart for enrollment and follow‐up. There were no study dropouts. The UP, the study treatment, focuses on identifying dysfunctional emotion regulation strategies common to all emotional disorders and helps patients tolerate and cope with emotions more adaptively (Barlow et al. 2011; Barlow et al. 2021). For the present study, a 12‐session, individual‐treatment format was conducted over a duration of approximately 3 months, with participants in the treatment group receiving weekly sessions. Participants completed measures at baseline, post‐treatment, and at 3‐month follow‐up.

### 2.1 Data Analysis

SPSS‐23 (Statistical Program for Social Science) was used to conduct study analyses. Descriptive analyses were conducted for the participants' socio‐demographicsocio‐demographic variables and the primary and secondary variables in which the effect of the intervention was examined. Depression and anxiety symptoms were considered primary outcomes. Group differences in pretreatment measures were first examined via an independent samples *t*‐test. A 2 (intervention‐control group) x 2 (pre‐posttest) mixed measures analysis of variance (ANOVA) was used to investigate the effectiveness of the UP in improving anxiety and depressive symptoms, increasing positive and decreasing negative affect, and improving psychological well‐being. To find the source of the difference in the scores, the Bonferroni test—onetest—one of the Post Hoc multiple comparison tests—was applied to compare the treatment outcomes between different time points, including pre and post treatment in the intervention and control groups. The statistical significance level was set at *p* < 0.05, while Hedge's g was used for effect size calculations, as done in previous studies (e.g., Farchione et al. 2012; Sauer‐Zavala et al. 2020). According to the classification proposed by Cohen (1988) to facilitate the interpretation of effect size values, 0.2 for small, 0.5 for medium, and 0.8 for large effect sizes. Moreover, changes in anxiety and depressive symptoms, positive and negative affect, and psychological well‐being during the intervention process were evaluated based on the Reliable Change Index (RCI) suggested by Jacobson and Truax (1991). The calculations required to obtain the RCI value were carried out using the online shared resource prepared by Chris Evans (https://www.psyctc.org/stats/rcsc.htm). If the absolute value of the calculated RCI exceeds the critical *z* score of 1.96 at the .050.05 significance level, the change is considered reliable; in other words, the participants has changed reliably after the intervention. In this study, RCI was used to examine the change at a individual level of each of the participants in the treatment group.

## 3 Results

### 3.1 Preliminary Analyses

There was no statistical difference between the two groups in terms of age, gender distribution, having a psychiatric diagnosis, or using psychiatric medication (*p* > 0.05). The results of the analyses are shown in Table 1. It was also found that there was no statistically significant difference between the mean scores of anxiety and depressive symptoms, positive and negative affect, and psychological well‐being variables between the groups (*p* > 0.05; see Table 2).

**Table 2:** Results of pre‐test comparison of treatment and control groups.

Variable	Groups	*n*	*M*	SD	*t*	*p*
Anxiety symptoms	UP	17	26.29	12.55		
	WLC	17	27.53	11.78	−0.30	0.77
Depressive symptoms	UP	17	25.88	9.55	−0.134	0.89
	WLC	17	26.35	10.91		
Positive affect	UP	17	22	5.66		
	WLC	17	25.94	5.78	−2.01	0.053
Negative affect	UP	17	30.23	8.32		
	WLC	17	33	8.15	−0.98	0.33
Well‐being	UP	17	31.18	11.54		
	WLC	17	28.70	12.37	0.60	0.55

Abbreviations: UP, Unified Protocol; WLC, Waitlist Control Group.

## 4 Effect of the Unified Protocol on Anxiety and Depressive Symptoms

The repeated measures ANOVAs revealed a significant main effect of group for anxiety and depressive symptoms (*p* < 0.05). Similarly, the main effect of time was found to be significant (*p* < 0.05). More importantly, the effect of the interaction between group and time was found to be statistically significant for anxiety (*F* (1, 32) = 17.65, *p* = 0.000, *η²* = 0.36) and depressive symptoms (*F* (1, 32) = 25.88, *p* = 0.000, *η²* = 0.45). The anxiety and depressive symptoms scores of the participants in the different groups in the pre‐test and post‐test significantly differed (see Table 3).

**Table 3:** Mean scores and standard deviations of study variables across conditions.

			Anxiety symptoms		Depressive symptoms		Positive affect		Negative affect		Psychological well‐being	
	*N*		*M*	SD	*M*	SD	*M*	SD	*M*	SD	*M*	SD
UP	17	Pre‐test	26.29	12.55	25.88	9.55	22	5.66	30.23	8.32	31.18	11.54
		Post‐test	10	9.97	8.82	6.18	33.12	8.21	16.53	5.42	43.41	8.94
WLC	17	Pre‐test	27.53	11.78	26.35	10.91	25.94	5.78	33	8.15	28.70	12.37
		Post‐test	27.88	15.10	25	11.49	23.59	7.01	31	7.05	30.88	11.70

Abbreviations: UP, Unified Protocol; WLC, Waitlist Control Group.

Following the post‐hoc comparison (Bonferroni), it was observed that the anxiety and depressive symptoms levels of the participants in the UP decreased after completing treatment. In contrast, there was no significant change in pre and post treatment measurements in the control group. According to the “Hedge's g” calculation (*Hedge g* = 1.75 & *Hedge g* = 1.46, respectively), it was then found that the effect size values had a large effect on the difference between groups at post treatment. Notably, the majority of participants (64.70%) in the treatment group demonstrated a reliable change in anxiety symptoms, while 76.47% of participants showed a reliable change in depressive symptoms.

To determine whether improvements were maintained, a paired sample *t*‐test was used, and the average scores obtained by the participants in the intervention group from the post‐test and 3‐month follow‐up tests were compared. There was no statistically significant difference between the scores from the post‐test to 3 months, indicating maintenance of the treatment effects (BAI: (*t* (16) = −0.23, *p* = 0.82; BDI: *t* (16) = −0.53, *p* = 0.61).

In addition to BDI and BAI, ODSIS and OASIS were used assess the effect of the intervention on severity of symptoms and impairment in functionality. ANOVA test for repeated measures was applied to compare the outcomes at pre and post treatment only in the treatment group. It was found that the mean scores of the measurements taken at pre and post treatment differed significantly (for ODSIS (*F* (1, 16) = 124.88, *p* < 0.05) and OASIS (*F* (1, 16) = 174.78, *p* < 0.05).

### 4.1 Effect of the Unified Protocol on Secondary Outcome Measures

Table 3 presents results for positive and negative affect and psychological well‐being variables as secondary outcome measures (i.e., PA, NA, PWB). The most notable finding is that the interaction effects of group with time were statistically significant (PA: *F* (1, 32) = 18.45, *p* = 0.000, *η²* = 0.37; NA: *F* (1, 32) = 19.60, *p* = 0.000, *η²* = 0.38; and PWB: *F* (1, 32) = 9.43, *p* = 0.004, *η²* = .0.23).

Bonferroni test indicated a significant reduction in dependent variable means from pre‐ to post‐treatment in the treatment group (*p* < 0.05). Moreover, the between‐group effect showed that the difference between the means of PA, NA, and PWB of the groups at post‐treatment was statistically significant (Hedges *g* = 1.25, 2.30, and 1.20, respectively). In addition, the percentage of participants who showed some improvement and reliable improvement, as well as no change and reliable deterioration, were calculated for the secondary outcome measurements in the treatment group. Accordingly, the percentages of participants who showed reliable improvement in PA, NA, and PWB were 64.70%, 70.59%, and 41.18%, respectively.

A dependent sample t‐test was conducted to investigate the maintenance of the intervention effects in the treatment group after 3 months. Accordingly, there was no statistically significant difference between the secondary outcomes from the post‐test to 3 months, (PA: *t* (16) = 1.33, *p* = 0.20; NA: *t* (16) = −2.04, *p* = 0.06 and PWB: *t* (16) = 1.51, *p* = .0.15).

## 5 Discussion

This study aimed to first culturally adapt the Unified Protocol for Transdiagnostic Treatment of Emotional Disorders into the Turkish language and then evaluate its effectiveness, primarily on anxiety and depressive symptoms, and secondarily, to preliminarily evaluate its effectiveness on positive and negative emotions and psychological well‐being. The preliminary findings of the current study should be interpreted within the following limitations inherent in this pilot study. First, the limited sample size makes it difficult to generalize our findings to a broader population. Second, the sample only included university students, and initial psychiatric conditions were not examined via structured clinical interviews. Finally, the study did not include an active treatment group as a control. Accordingly, the present findings should be replicated with larger samples, inclusion of diagnostic interviews and active control grups. Nevertheless, despite these limitations, this is the first study in which the UP was adapted to Turkish, and its effectiveness was evaluated comparatively with 3‐month follow‐up data.

Before moving on to the main findings, another striking element in the sample was the absence of dropouts that is none of these participants withdrew before completing the treatment or waitlist period. From one point of view, this is consistent with findings in the relevant literature that the dropout rate in the UP is relatively low, a feature that may distinguish it from other standard CBT protocols (Ellard et al. 2010). On the other hand, a similar condition in the waitlist group (i.e., the absence of dropouts) also reminds us to consider the possibility that dropouts did not occur due to the nature study sample. Given that the sample consisted of university students, this group is less likely to seek psychological support elsewhere due to lack of financial and other practical conditions, although they may have needed to get. Therefore, participants may have considered waiting for treatment worthwhile.

As for the findings, the Turkish UP reduced anxiety and depressive symptoms in the treatment group compared with the control group. It can be reported that this is the first study supporting the use of the Turkish version of the UP as an effective manual in reducing anxiety and depressive symptoms. These results are consistent with previous studies (Ellard et al. 2010; Farchione et al. 2012; Osma et al. 2015; and Ito et al. 2015) and provide additional support for the ecological validity of using the UP for emotional problems.

The UP—which is efficacious for improving anxiety and depressive symptoms—may also provide additional benefits such as enhanced positive affect and psychological well‐being. Our findings detected significant increases in positive affect and psychological well‐being levels and significant decreases in negative affect in participants who received the UP compared to their pre‐test measurements. Moreover, when compared to the control condition at posttreatment, we found that positive affect and psychological well‐being scores were significantly higher, and negative affect significantly lower, in the intervention group. These findings suggest that the emotion regulation skills addressed in the UP may significantly change affect. Similar findings were also reported in efficacy studies of the individually‐administered UP. For example, while Ellard et al. (2010) and Sauer‐Zavala et al. (2012) specifically reported a reduction in negative affect, Spencer‐Laitt et al. (2023) emphasized an increase in positive affect, especially in joviality. All of these findings suggest that the UP not only reduces the frequency and intensity of negative affect but also increases the frequency and intensity of positive affect.

The results of this study also support the durability of the intervention over 3 months. Several studies on the UP have found that improvements in anxiety and depression levels continue even beyond 6 months (Barlow et al. 2017; Farchione et al. 2012; Eustis et al. 2020), while other studies have shown that treatment effects are sustained over longer periods (Bullis et al. 2014; Eustis et al. 2020; Bullis et al. 2023). In sum, all of the findings from this study are consistent with the findings of both the research team that developed the UP (Ellard et al. 2010; Farchione et al. 2012) and the researchers who had adapted the UP to different cultures (e.g., Spain, Osma et al. 2018; Japan, Ito et al. 2015; Iran, Mohammadpour et al. 2018). Thus, this study also provides additional support regarding the cross‐cultural validity of the UP.

There are several clinical advantages associated with the UP. The treatment's ability to simultaneously address comorbidity allows clinicians to address a range of the most commonly occurring emotional disorders with a single protocol consisting of five core treatment elements in less time. In addition, according to the feedback from the participants, the most beneficial module during the application phase was the exposure exercises. On the other hand, it was observed that some participants did not need exercises involving exposure to physical sensations. Similar feedback was also revealed in a study evaluating the implementation of UP in a group format in Spain (Osma et al. 2015). Particularly, participants suffering from depressive symptoms did not find it meaningful to do these exercises. All participants were asked to complete at least one physical sensation exposure exercise, with the rationale that they would benefit from these exercises in the future if needed. Apart from this, the participants stated that they found all the practices in the modules necessary and meaningful. Having the materials ready, the weekly program clear, and the protocol containing many techniques enabled the therapist to carry out the process easily. The ODSIS and OASIS were used as a therapeutic tool for the participants to observe themselves during the treatment. Each week, these measurements helped the participants gain awareness of their ups and downs. The therapist received supervision from second author for any negative effects that might occur and situations that might be overlooked, and the therapist's emotions and difficulties were addressed at regular intervals.

Providing an alternative to multiple single‐disorder protocols, UP makes training clinicians in evidence‐based practice easier, thus addressing a significant need in Turkey through increased access to mental health services. Results of this study support the assumption that the UP can be effectively implemented in units where preventive interventions are especially important, such as primary health care services or university health centers. Moreover, considering that most of the studies on the effectiveness of the UP in the treatment of various psychological disorders—especially anxiety disorders and depression—have been conducted in Western countries, it can be said that this study contributes to the literature on the generalizability of the UP to different cultures. Consequently, the findings regarding the effectiveness of the Turkish UP were consistent with the ones obtained as a result of examining the adaptation and effectiveness of the protocol to different cultures (e.g., Ito et al. 2015; Osma et al. 2018; Castro‐Camacho et al. 2023). This study has taken the first step towards assessing the use, effectiveness, and acceptability of the UP in Turkey, which is relevant for increasing treatment accessibility. Future studies on the Turkish UP adaptation should be performed to find additional reliability in the cultural adaptation, including with a different age demographic and a larger sample size. Finally, considering the nature of present study, assessment of treatment adherence and competence is also critical issue for future studies.

## Ethics Statement

Ethical approval for this study was obtained from ethics committee of Dokuz Eylul University.

## Conflicts of Interest

The authors declare no conflicts of interest.

## Data Availability

The data that support the findings of this study are available on request from the corresponding author. The data are not publicly available due to privacy or ethical restrictions. The data of the findings in this study are available from the corresponding author upon any request.
